# Health technology design considerations specific to Indigenous Data Sovereignty and implementation science

**DOI:** 10.3389/frhs.2026.1731352

**Published:** 2026-05-07

**Authors:** Alec J. Calac, Tiana McMann, Joseph Yracheta, Tim K. Mackey

**Affiliations:** 1 UC San Diego School of Medicine, San Diego, CA, United States; 2 Global Health Policy and Data Institute, San Diego, CA, United States; 3 S-3 Research, San Diego, CA, United States; 4 Native BioData Consortium, Eagle Butte, SD, United States

**Keywords:** blockchain technology, digital health, framework, genomics, implementation, science, Indigenous

## Abstract

There is growing attention to the importance of Indigenous Data Sovereignty (IDS) in the practice of medicine and public health, especially in the United States. Through a multi-year research collaborative between the University of California, San Diego and the Native BioData Consortium, it became clear that the use of an implementation science framework might guide the design of emerging digital health technologies best respecting the interests and priorities of Indigenous Peoples. In this Case Study, we discuss the integration of the Consolidated Framework for Implementation Research (CFIR) and Expert Recommendations for Implementing Change (ERIC) to inform the design of policies, programs, and interventions focused on improving Indigenous health. We explore the movement for IDS from the perspective of contextual concerns raised by Indigenous Peoples, researchers, and their allies specific to the design and implementation of digital technologies primarily in a North American context. We then explain how CFIR, ERIC, and similar resources can be used to design digital health technologies for use in Indigenous contexts.

## Introduction

New insights into human disease, genomics, and precise identification of individual- and community-prevalent gene variants with clinical significance have greatly benefited the practice of medicine (1–3). Concomitantly, the health and economic benefits of digital technologies, including machine learning, artificial intelligence, next-generation genomic sequencing systems (NGSS), and distributed ledger technologies are slowly being realized in under resourced communities (4–6). Working to design these technologies in partnership with community leaders will help engage geographically isolated communities and those individuals hesitant to submit their health data to external biorepositories (4–6).

However, in the United States (U.S.), the use of these new technologies may outpace the development of responsible use guidelines in American Indian and Alaska Native (AI/AN) communities, one of the most geographically heterogenous Indigenous populations, with legal protections derived from treaties signed with the federal government, and other minority groups (7–12). In 2019, the National Institutes of Health All of Us Research Program, in an effort to increase the representation of underrepresented groups in health databases, convened an AI/AN Advisory Group to identify appropriate practices for engaging AI/AN communities in precision medicine and NGSS-enabled research, but there were concerns that this did not constitute sufficient community consultation with AI/AN governments and their members (13, 14).

While the All of Us AI/AN Advisory Committee published some considerations for responsible management of AI/AN health data, they did not detail equitable benefit sharing among participating communities or safeguards against the commodification or sale of AI/AN genomic data by non-AI/AN individuals and entities involved in the design and operation of technologies interfacing with AI/AN communities (15–19). Whether explicit or not, this type of commodification reifies the legacy of extraction from AI/AN communities, whether related to cultural knowledge, genomic profiles, natural resources, or economic capital. This underscores the sociopolitical importance of the concept of Indigenous Data Sovereignty (IDS), the right of an [Indigenous] nation to govern the collection, ownership, and application of its own data (11, 20, 21), especially when AI/AN individuals, organizations, and governments are not always aware of how their data are managed and used by external parties (22).

Critically, there is a need to emphasize the importance of IDS in the application of principles and frameworks specific to data governance and management (e.g., the FAIR and Open Data Principles that have not undergone consensus agreement processes or development with AI/AN groups) in these settings. One specific case study that exemplifies the need for IDS and tribal oversight is genomics-focused NGSS and related digital health technologies that seek to promote health equity among AI/AN communities.

In response, this Community Case Study aims to explore the movement for IDS from the perspective of contextual concerns raised by AI/AN community members, researchers, and their allies specific to the design and implementation of digital technologies primarily in a North American context. The second aim of this article is to serve as a resource for audiences interested in engaging Indigenous partners in the conduct of research and technology development aligned with IDS principles. Each sub-section will have varying relevance to academics, decision-makers, and Indigenous knowledge holders with interests in advancing the health and wellness of Indigenous Peoples. For the purposes of this discussion, we use the term “AI/AN” and “tribal” when discussing the Indigenous context specific to the U.S. (e.g., community values, applicable laws and regulations, race, ethnicity, and sovereignty discourse), but intend for the lessons learned to be applicable to all Indigenous contexts across the globe. The third aim is to explain and describe how Implementation Science can be used to design digital health technologies for use in AI/AN and other Indigenous contexts and how it comports with consensus-driven decision-making and cultural norms in these communities.

The motivation for this Case Study originated from an earlier project where co-authors sought to identify technology-enabled features mediated by blockchain technology (a decentralized digital ledger that provides immutable record-keeping and real-time verification of user authority) that could operationalize IDS in partnership with the Native BioData Consortium (NBDC), the first AI/AN-led biorepository on tribal lands (23, 24). Through a series of conference presentations and engagements with the project's advisory board, it became clear that the design of this system would extend far beyond the project objectives and may benefit from the use of an Implementation Science framework to better identify barriers and facilitators of successful implementation that could also inform future projects involving technology and Indigenous peoples.

## Materials, methods, context

### Key theoretical foundation for study

**Figure 1:**
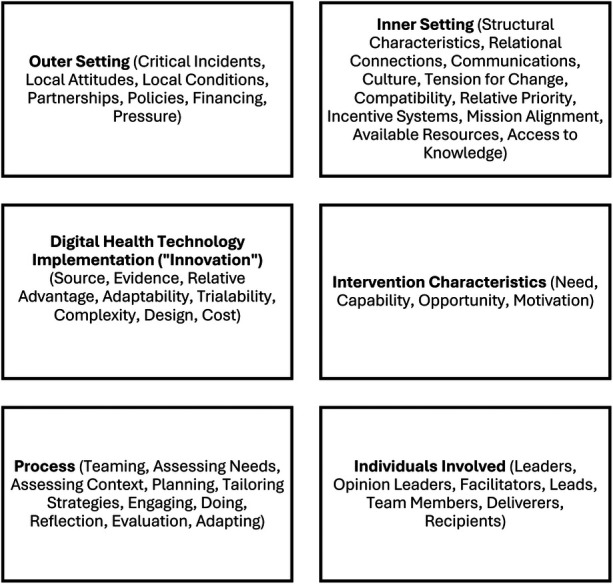
Adapted consolidated framework for implementation research (CFIR) 2.0 levels and associated measures: innovation, inner setting, outer setting, intervention characteristics, process, and individuals involved. The Outer and Inner Settings are the underlying context for the implementation of the Intervention. The Innovation and Intervention Characteristics are mediated by the Process and Individuals Involved.

The Consolidated Framework for Implementation Research (CFIR) is a multi-level framework that considers the characteristics of the digital health technology being implemented (innovation), outer setting (e.g., federal rules and regulations), inner setting (e.g., available resources), individuals involved (e.g., engineers, community members, technical advisors), and design process (e.g., needs assessments, interviews, iterative evaluation) (25). Each CFIR level has several associated constructs which are detailed in Figure 1 and defined for practitioner use for adaptation to local contexts here (https://cfirguide.org/constructs/). Adaptation for use should first begin with operational definitions of each CFIR level and associated measures then iteratively changed as information is collected from key figures involved in technology and program development. The dynamic nature of these definitions also reflects the heterogeneity of perspectives within and between AI/AN and other Indigenous communities.

### Development of CFIR-ERIC framework for IDS in digital health

Using the CFIR framework as its underpinning construct, this Community Case Study considers which CFIR 2.0 measures have relevance specific to IDS and digital technologies and then presents a set of Expert Recommendations for Implementing Change (ERIC) strategies generated from the publicly available CFIR-ERIC Matching Tool (https://cfirguide.org/choosing-strategies/). This tool tailors strategies to the anticipated implementation barriers for an innovation across the six CFIR domains. These strategies are specific to our described implementation of digital health technologies in a U.S.-based context, but may have applicability in other settings.

The organization of this Case Study is based on topics and themes frequently identified in research and education frameworks as barriers and facilitators to IDS as identified in the literature, such as reciprocity and benefits sharing, authority to control, and data system interoperability (22, 26, 27). These frameworks include: The Four R's (Respect, Relevance, Reciprocity, and Responsibility), CARE Principles for Indigenous Data Governance, and FAIR Principles. The first set of principles are meant to promote the retainment and advancement of Indigenous students in higher education but do have relevance and applicability to “big data” when we consider “data as a relation” or as an extension of oneself (26). This leads into two other frameworks, the CARE Principles for Indigenous Data Governance, which detail data governance considerations for Collective Benefit, Authority to Control, Responsibility, and Ethics, and the technology-focused FAIR Principles, which posit that data must be Findable, Accessible, Interoperable, and Reusable by the individuals and organizations providing these data (22, 27). The constructs in these frameworks all have relevance to IDS and CFIR constructs and may also have policy implications as will be discussed.

### Adapting an implementation framework for IDS, digital technology, and implementation science

With 575 federally recognized AI/AN Tribes and Villages, implementation of any digital health technology or innovation needs to be mindful of local contexts and respect community norms and values. It is likely that CFIR levels (Inner Setting vs. Outer Setting) will have varying importance between different AI/AN communities. Thus, it will be important to identify the right individuals and groups responsive to the constructs of each CFIR domain, because there is no one individual or party that can provide the information necessary to address all implementation domains described in the framework.

To help guide technology implementation considerations in AI/AN and other Indigenous contexts, one may consider the relative importance of each CFIR construct. Successful implementation of a digital health technology will require consideration of its: (1) Adaptability *(*Intervention Characteristics Domain), which may be described as the degree to which a digital health technology system is adapted or “fit-for-purpose” (designed to be customizable and modular to community needs) (28); (2) Cost (Intervention Characteristics Domain) as the anticipated cultural implications, environmental impact, and economic capital associated with the digital health technology (29), (3) Context (Process Domain) as the detailed steps (with associated rationale) that have been taken facilitate co-governance of digital health technologies with Indigenous leaders; and (4) Incentive Systems [Structure] (Inner Setting Domain) as the economic-derived or preferred form of benefit for individuals contributing potentially sensitive data to a digital health technology system (22).

Early and sustained engagement with community members throughout the design process may also provide an opportunity to assess Compatibility, Mission Alignment, and Relative Priority (Inner Setting Domain of CFIR) with community members and Indigenous-serving organizations to determine if a proposed digital health technology can address an identified community need and is seen as a priority relative to other ongoing or planned internal and external initiatives. Related facets of implementation relevant to IDS and successful implementation are culture, cost and responsible stewardship of the environment. Specifically, consideration of how Indigenous Peoples have responsibly stewarded their culture and natural environments since time immemorial needs to be taken into account. Notably, culture is a named measure in the Inner Setting of CFIR, demonstrating its significance in the implementation process. Thus, when engaging AI/AN communities it is important to demonstrate an understanding of that relationality to the natural and built worlds.

Hence, this study also outlines an implementation science methodology interpreted from CFIR measures linked to IDS for digital health technology development.

## Results

### IDS core principles applied to health technology development

Based on results from the development of the CFIR-ERIC framework for IDS and digital health, here we detail specific IDS principles aligned with CFIR measures relevant to the development of health technologies. These include specific sub-themes of IDS as a determinant of Indigenous Health, responsible engagement of technology design with tribal communities, alignment of value propositions and ensuring equitable benefits sharing for tribally directed technologies, capacity building and respect for Indigenous perspectives, consideration of data ownership and creative and intellectual property rights, and the incorporation of digital technologies into public health practice.

Key to IDS is the fundamental principle of ensuring responsible use of Indigenous data and related technologies. Indigenous and decolonial scholars have long posited that there are many facets of data that can be explored at the intersection of data science and Indigenous epistemologies (30). For example, framing of “data as a relation” purposefully incorporates culture and relationality as two of many possible considerations in the design and implementation of AI/AN-focused digital health technologies (31). It also welcomes co-ideation and co-creation of using these technologies for research with data scientists, AI/AN leaders, and interested parties to strengthen trust, increase internal capacity, and foster collaboration, which are greatly relevant to the Inner Setting, Outer Setting, and Process Domains of CFIR (32). This might even involve allies and partner institutions advocating for funding designs and application review processes that respect community values and priorities. Responsible collection and use of data also honors how Indigenous Peoples have stewarded their cultures and traditions since time immemorial.

### Indigenous Data Sovereignty as a determinant of indigenous health

External, non-Indigenous governmental policies, rules, regulations, and laws may influence Indigenous health and affect how and when IDS is exercised, often without deference to Indigenous leaders and governing bodies (33). Another challenge to promoting health equity in AI/AN and other Indigenous communities is varying recognition of their sovereignty by heads-of-state and their respective settler governments. For example, even when an AI/AN governmental entity is recognized by the U.S. government, their free exercise of IDS and other affairs is limited to the geographic borders of their respective land bases, unless otherwise specified by applicable U.S. statutes (e.g., Public Law 93-638, Native American Graves Protection and Repatriation Act) and judicial proceedings (*McGirt v. Oklahoma, 2020*) (12). In the digital era, a key facet of IDS is not just the legal authority of an AI/AN or Indigenous entity to regulate data management and research conduct, but also collaboration and shared jurisdiction with public health officials and other agencies handling data that may have implications for Indigenous governance (34). Data control and research parameter management might be the impetus to developing new regulations and protocols within the network of governmental agencies consulting with Indigenous public health authorities. This support is crucial, especially during times of public health crisis (e.g., pandemic preparedness and response, occupational exposures, disease surveillance), because not all communities have the capacity or infrastructure to carry out these functions without external assistance (35).

In the U.S., there were numerous impediments to IDS during the COVID-19 pandemic, such as misclassified AI/AN mortality data, limited resources, and inadequate data-sharing infrastructure between local, state, tribal, and federal public health systems (36, 37). These challenges, largely due to limited engagement and co-management of data systems with tribal public health authorities, led to a tremendous loss of life among AI/AN communities, which could have been avoided (38). Hence, public health practitioners interested in health information technology modernization and working with tribal public health authorities may consider embracing health technology design processes that prioritize IDS and end-users' (individuals' and communities') needs and priorities in the design of novel technologies (39). The product of these collaborations may to lead to more robust public health responses in AI/AN communities, increase the quality of data provided to tribal public health authorities (e.g., Tribal Epidemiology Centers), and opportunities for targeted investment of resources from the Indian Health Service, Substance Use and Mental Health Services Administration, Centers for Disease Control and Prevention, and other U.S. federal agencies interfacing with AI/AN Tribes and Villages.

### Responsible community engagement in technology design

Technologies for Indigenous communities should not be designed without input from AI/AN stakeholders, including elected leaders, health officials, elders, and/or community members. The input-gathering process may extend research and engineering timelines but helps prioritize Tribal self-determination and self-governance over the interests of non-AI/AN stakeholders. When engaging with community members and proposing a new project or innovation, it is important to describe how the innovation will materially or immaterially benefit AI/AN individuals and communities and to identify the research groups' positionality to the proposed work and the community being engaged with, and how such a partnership can be mutually beneficial to all parties involved. Reciprocal relationships and fostering a culture of transparency facilitates opportunities for co-creation or co-design of technologies with members of the community to facilitate AI/AN and Indigenous data governance (40). It also provides space for community members and leaders to voice their concerns during the earliest stages of collaborative design processes when systems are most amenable to change (e.g., adjusting based on pilot projects that fail to meet community-focused project goals). Further, any engagement or co-design process involving AI/AN communities should be primarily exploratory, with no preconceived determinations about the design or choice of a technology system, including the type of managed data (39). Instead, a “menu” of options should be provided to AI/AN stakeholders to increase their agency in the design process and highlight existing uses for a proposed technology in other AI/AN or Indigenous contexts, if available, and how these systems and interventions may be adapted in real-time.

### Capacity building and respect for indigenous perspectives

It is important to involve Indigenous Peoples not just as collaborators or community partners, but also as active members and leaders of the implementation team, ranging from champions for implementation within their community to designers and evaluators of the implementation itself. This includes being involved in the conduct of public health research and related activities, because it helps increase local capacity and fosters positive relationships. Indigenous individuals involved in an implementation will likely be familiar with constructs outlined in the Outer and Inner Setting Domains of CFIR (e.g., local attitudes, tension for change) and knowledge about the different policy tools and regulatory mechanisms for engaging sovereign Indigenous governments and their associated entities (41, 42).

Co-creation between engineers, data scientists, and Indigenous Peoples is likely to foster the exchange of invaluable skills and knowledge, including specialized training in data systems management and opportunities to discuss cultural perspectives on health and wellness. Indigenous team members can also advise the creation of culturally-appropriate data transfer ownership and use agreements respecting IDS and free, prior and informed consent outlined in the non-legally binding United Nations Declaration on the Rights of Indigenous People (UNDRIP), which has limited applicability in the U.S. (subject to constraints of AI/AN sovereignty by federal law) and greater relevance in global Indigenous contexts (43, 44).

In the U.S., the exercise of IDS is mainly facilitated by the Indian Self-Determination and Education Assistance Act of 1975 (Public Law 93-638), which enables AI/AN Tribes and Villages to manage programs and services involving their members on their own lands. Implementation facilitators can also encourage the adoption of agreements (e.g., Memorandum of Understanding) that operationalize IDS by preferencing the interests of Indigenous communities over the interests of external stakeholders, which can build a culture of reciprocity and mutual benefit with AI/AN and Indigenous Nations. These agreements are of particular utility for AI/AN Tribes and Villages who lack or are seeking federal recognition by the U.S. government, but still wish to be involved in program, service, and technology governance and operation.

Tribes may also consider the use of technical advisors to facilitate aspects of health technology design processes that are beyond their understanding and convey reasonable expectations to partners, such as honoring community values and respecting IDS. In the CFIR model, technical advisors might be considered individuals involved with the implementation who function to improve internal capacity and AI/AN organizational readiness for change. While there are efforts to train an AI/AN data science and genomics workforce, such as the Summer Internship for Indigenous Peoples in Genomics (SING) Program and IndigiData Conference, the goals of these initiatives will likely not be fully realized for a generation (45). In the interim, Tribal leaders continue to rely on technical advisors and AI/AN and Indigenous-serving organizations to establish and maintain relationships with external partners and allies to build capacity and benefit from emerging health technologies, such as NGSS.

Ultimately, bringing diverse Indigenous perspectives to health technology design and implementation teams, including those focused on NGSS, may highlight the unique challenges that Indigenous Peoples face in their respective countries when conducting biomedical, behavioral, and social sciences research. Further, their involvement may lead to the identification of issues and challenges that are not readily apparent to non-Indigenous members of the implementation team, such as the potential for cultural and environmental harm associated with technology design and operation. Indigenous team members may also be able to identify technology use cases appropriate (or inappropriate) for their local contexts and describe how digital resources and distributed tools like digital identification wallets (identity verification) and dynamic consent mechanisms can respect the rights and privacy of Indigenous communities and operationalize IDS for the benefit of all (46, 47). In addition to proactively identifying concerns with potential use cases, such as genomics, they may also be able to identify solutions or alternative approaches that mitigate ethical, social, and cultural concerns. For example, if environmental harm is a concern, the implementation team could work with community leaders to identify strategies to minimize the environmental footprint of energy-demanding data systems, emphasizing the importance of interdisciplinary teams and community engagement (48, 49).

### Value propositions and equitable benefits-sharing for technology

Data from Indigenous Peoples is more than an asset or resource. It should be viewed as an extension of the individual with an inherent obligation to safeguard it across its lifecycle. In the era of big data and open-data environments, Indigenous leaders and communities may also wonder if digital health data have use cases that extend beyond research and cultural revitalization, such as commercialization and commodification. It is likely that peoples' attitudes and beliefs will vary on these subjects, such as acknowledging the potential risk of loss of confidentiality and stereotyping of AI/AN individuals based on their genetic information. Tribal governments should come to a consensus as to what types of benefits they would like to be returned to their communities and specify these requirements in legally-binding agreements that uphold IDS principles.

Equally, Tribal leaders, AI/AN public health officials, and respected community figures, such as elders and knowledge holders (Inner Setting Domain of CFIR), may wonder whether digital health technologies have resource-priority, value, or even perceived benefit in AI/AN communities, especially when so many AI/AN households and communities lack basic necessities, such as electricity, access to high-speed Internet, and running water (50). Privacy is also a concern as Tribal communities range in size from the hundreds to hundreds of thousands. The use of digital health technologies may even widen the equity gap and digital divide between low-resourced and well-resourced AI/AN communities given differences in population size, economic resources, and infrastructure. For example, mathematical challenges associated with computational genomics of Mendelian cohorts or culturally isolated populations (e.g., Tay-Sachs families or Amish families to which Tribes bear similar genetic isolation) can be more appropriately informed by tribally-directed technologies and new computational informatics tools and approaches (e.g., multi-omics analysis, deep phenotyping, computational prediction, etc.) that need contextual background to understand the context of data provided by these communities (51–53).

Further, the establishment of Inter-tribal consortia, and identification of external change agents and champions, to help facilitate health technology implementation may help bring benefit to AI/AN communities who might not otherwise have the capital or infrastructure to do so. Policymakers and technology implementers may also consider establishing regional pilot programs to identify barriers and facilitators specific to small, medium, and large AI/AN communities. Tribally-informed technologies may also address computational problems and maintain tribal privacy and anonymity, avoiding stereotyping and stigmatization as well as tracking group ownership and provenance.

### Consideration of data ownership and intellectual property

Tribes' reliance on external partners and academic institutions to design and operate digital technologies, develop novel algorithms, and analyze health data warrants a discussion about technology and data ownership, technology transfer, and creative expressions and intellectual property rights (54). While there is benefit in forming external and tribal-academic partnerships (from a capacity-building perspective), there may be conflicts between IDS principles, funding agencies, and research institutions in determining ownership of health information technology infrastructure components (Innovation Characteristics Domain of CFIR) using data generated by AI/AN individuals and communities (55). This may also be complicated by ownership specifications delineated by a Tribal IRB or research review board in data management and data use agreements (56). As IRBs often are external and limited in their legal or regulatory authority (they apply to federally funded research projects and have no authority over tribal entities), Tribal governments often establish intra- and inter-tribal systems of governance that go beyond traditional scientific ethics for data tracking when geographically, culturally or genetically close tribal nations can be exploited as the proxy for their regional neighbors (55, 56). Through principles such as Canada's OCAP™ (tribal ownership, control, access and possession) and recognition that genetics and culture overlap across landscapes Tribes can begin to collectivize and jointly track potential Intellectual Property that two or more tribes may have in common. Further, the potential for tribally-directed and managed data ownership, rights, and benefit sharing systems, such as blockchain technologies that can enable decentralized, immutable, and tamper proof digital ledgers for recording IP contributions and assets (e.g., facilitation of tribal digital rights management), could offer opportunities to operationalize IDS as has been proposed in other Indigenous data management contexts (24, 57).

### Incorporation of digital technologies into public health practice

Primary considerations for Tribal public health authorities interested in using digital health technologies should assess structural characteristics, such as physical infrastructure (e.g., facility cost, land management) and information technology infrastructure (e.g., data system capabilities) (Inner Setting Domain of CFIR), as to whether at present, or with investment, the innovation is possible. It also needs to be determined if the tribal public health or data systems entity has the information technology infrastructure and technical expertise among personnel to operate and maintain digital health technologies. The data and insights generated by these technologies should also have social, cultural, and/or economic relevance to community members (Inner Setting Domain of CFIR) and be able to be integrated into interoperable clinical, public health, and social services' data systems infrastructure. Interoperability can help maximize the benefit derived from digital health technologies and promote the usability of data across different business sectors, which in turn promotes IDS.

## Constructing an implementation science methodology for IDS and digital health

Acknowledging the heterogeneity of Indigenous cultures and norms, we provide our own interpretation of how CFIR measures can link to IDS for technology development, which may vary from community to community. IDS principles have relevance to the CFIR constructs outlined in Figure 1. The design process and application of these principles to different types of digital health technologies (e.g., artificial intelligence applications, blockchain-based tools, federated learning approaches, NGSS) highlights several CFIR constructs with relevance to AI/AN and other Indigenous contexts. In this Case Study, they were considered anticipated barriers to the successful design and operation of a digital health technology in an AI/AN community. These constructs fall under the **Intervention Characteristics** (Adaptability, Cost), **Inner Setting** (Culture, Organizational Incentives, Relative Priority, Structural Characteristics, Available Resources, Compatibility), **Outer Setting** (Partnerships, Policies and Laws), and **Process Domains** (Champions, External Change Agents).

Beginning with Intervention Characteristics, early engagement with tribal leaders in AI/AN communities in the design process allows for their values to be embedded in the initial technology prototype within allowable costs and adaptability for their stated use cases. In our blockchain use case, engagement with the Community Advisory Board (tribal members in the Great Plains region of the U.S.) and prior tribal leader input to the NBDC set the scope and defined uses of the blockchain system for the engineering team. This process of co-creation facilitates a link between the Inner Setting and Outer Setting by allowing for AI/AN and Indigenous communities to exercise their inherent right to free, prior and informed consent before outside partners seek resources and personnel to implement new health technologies. It is also important to note that policies and laws in the Outer Setting (outside of Indigenous contexts) may conflict with those of the involved Indigenous communities, which highlights an ongoing need for advocacy and greater recognition of how Indigenous Data Sovereignty may be constrained in certain settings (a function of the Process Domain involving Local Champions and External Change Agents), such as the domestic-dependent relationship between American Indian and Alaska Native Tribes and Villages and the U.S. government.

By identifying these barriers ahead of formal implementation, the public CFIR-ERIC Matching Tool (https://cfirguide.org/choosing-strategies/) can be used to identify evidence-based strategies specific to these barriers and promote successful implementation of the technology-of-interest. The CFIR-ERIC Matching Tool reports the percent relevance of each construct to an ERIC strategy and sorts the strategies in descending sum percent relevance. Discussion notes from meetings with the Community Advisory Board were reviewed by all members of the UCSD-NBDC Research Collaborative, which then identified any CFIR-ERIC constructs that had potential alignment with perceived implementation barriers by the Advisory Board. A section of each of these meetings focused on the use of open-ended questions to elicit members' perspectives on digital health technologies, including their prior understanding of these systems, which were then compared to the available CFIR constructs. The eleven constructs identified by a majority consensus of the Research Collaborative were used to identify the ten most relevant CFIR-ERIC strategies for the implementation of the proposed blockchain health system, are listed in Table 1.

**Table 1:** Implementation strategies for hypothetical CFIR barriers using the CFIR-ERIC matching tool.

Matched CFIR-ERIC Strategies	Perceived CFIR-ERIC Barriers and Identifying Constructs	Strategy Evaluation
Identify and prepare champions	Culture Opinion Leaders (e.g., Elders) Champions (e.g., Advocates, Political Leaders)	Qualitative and Quantitative Methods
Assess for readiness and identify barriers and facilitators	Culture	
Build a coalition	External Change Agents (e.g., Policymakers)	
Conduct local consensus discussions	Relative Priority (ie., Individual vs. Tribal)	
Alter incentive/allowance structures	Organizational Incentive and Rewards	
Promote adaptability	Adaptability (ie., Fit-For-Purpose)	
Inform local opinion leaders	Opinion Leaders	
Access new funding	Cost (Cultural, Economic, and Environmental) Available Resources	
Identify early adopters	Opinion Leaders	
Tailor strategies	Compatibility (Sociocultural and Technological)	

Future work with the UCSD-NBDC Research Collaborative involves dissemination of the project findings to AI/AN researchers and community practitioners as well as implementation, validation, and evaluation of the matched CFIR-ERIC strategies to implement blockchain-based governance systems protecting sensitive health data. The evaluation of this framework is likely to favor qualitative over quantitative methodologies, including photovoice (narrative storytelling using photography and other imagery), focus groups, and mixed-methods triangulation to integrate and co-validate Western and Indigenous perspectives regarding design success.

## Discussion

These IDS core principles and implementation science method strategies, along with others available in the CFIR-ERIC Matching Tool, have the potential to guide and facilitate the successful implementation of digital health technologies for the purposes of operationalizing IDS to benefit Indigenous communities. The ten strategies listed function across all CFIR domains and should be incorporated into operational design plans and workflows, ensuring that each is properly addressed. They primarily focus in the CFIR Inner Context or the setting in which the technology is implemented (see again Figure 1).

Building consensus is an important strategy for any proposed intervention or innovation involving AI/AN communities. Ideally, researchers and members of the implementation team should first engage with AI/AN leaders to understand local priorities, then start to engage with AI/AN health systems leaders to better understand the issues and challenges faced by the community, and eventually design a system tailored to those needs. The digital health technology system can then be presented at community forums to further understand what needs this technology should address and what hesitations members of the community have about the implementation process or technology itself. As discussed earlier, building consensus and purposefully including community feedback into the technology design will ensure that the technology respects the preferences of the AI/AN community and their reasonable expectation to critical aspects such as privacy and benefits sharing as outlined in IDS. This strategy is also closely related to the strategy for identifying and preparing implementation champions. These champions, likely to be members of the participating AI/AN community, can be an interface between the implementation team and members of the community to share concerns, feedback, and other important information that can promote successful implementation of digital health technology systems. The use of champions also promotes IDS because community voices are central to the decision-making and design and ensures that technologies respect local norms and regulations.

While this Case Study primarily focused on design considerations for AI/AN communities with recognized governance and sovereignty over matters involving their members and lands, the free exercise of IDS away from tribal lands is also of great importance for urban AI/AN residents and non-recognized or state-recognized AI/AN governments interested in safeguarding their data and privacy without the explicit protections available to federally-recognized AI/AN governments. New funding streams and support from local and state officials, as suggested by the CFIR-ERIC Matching Tool, may also support the development of infrastructure and systems that can address these issues and identify design considerations that protect urban AI/AN residents (58).

Finally, it may be important to focus resources and attention towards other domains, such as the Outer Setting (structural and societal factors), Intervention Characteristics (evidence, acceptability), and Implementation Process (timeline, resources) (59). All CFIR-ERIC strategies have a strong evidence base for promoting positive intervention and implementation outcomes (60). Non-Indigenous stakeholders and allies such as academic institutions, non-profit organizations, and local governments should also be considered as potential barriers and facilitators when considering these strategies, given the close relationship between the Inner and Outer Setting Domains of CFIR. It is also important to identify and prepare champions within the community that can garner positive support for the implementation, identify incentives to encourage community participation and implementation use/uptake, and incorporate cultural knowledge and values into the design and implementation of novel health technologies.

In follow-up research studies in other AI/AN or Indigenous contexts, use of focus group participants (which can be adopted to existing Indigenous contexts like Sharing Circles), community listening sessions, public deliberation, and talking to tribal-specific key informants may identify other perceived barriers to implementing digital health technologies in their own respective and diverse communities (61–65). Evaluating the effectiveness of these strategies with Western and Indigenous methodologies will also support a robust, IDS-centric implementation and recognize the importance co-design approaches for technologies with Indigenous partners (66, 67).

## Conclusions and acknowledgements

Community consultation and engagement is paramount to promoting health and data equity in AI/AN communities, not just at the federal level but especially when implementing technologies and programs that engage local community members (53). The reflections and proposed implementation strategies presented in this Case Study are likely to support the ongoing operationalization of IDS in research, clinical, and public health practice (68). The identified ERIC strategies align well with recommendations that have been developed for operationalizing IDS in health-focused research and other settings (14, 42, 69). Future consideration should be given to the AI/AN-specific ethical, legal, and social factors that function as barriers and facilitators for the implementation of emerging digital health technologies, and how tools like CFIR-ERIC can facilitate these processes (70). Many of the strategies identified in Table 1 highlight the importance of shared data governance and engaging all members of an Indigenous community in the design and implementation stage, ranging from building consensus to promoting adaptability (“fit-for-context”) and tailoring implementation strategies (71).

There is certainly a paucity of literature using CFIR-ERIC in Indigenous contexts and we hope that others will use this tool in their own collaborates to help guide policy, program, and health technology design. We hope this Case Study provides a roadmap for responsible engagement with Indigenous communities when developing digital health technologies, which when appropriately incorporating implementation science constructs and honoring IDS principles, should foster technology that is responsive to the real-world needs and values of Indigenous peoples while supporting their right to self-governance and self-determination.

## Data Availability

The original contributions presented in the study are included in the article/Supplementary Material, further inquiries can be directed to the corresponding authors.

## References

[1] Shendure J Findlay GM Snyder MW. Genomic medicine–progress, pitfalls, and promise. Cell. (2019) 177:45–57. 10.1016/j.cell.2019.02.003 30901547 PMC6531313

[2] Evans W Meslin EM Kai J Qureshi N. Precision medicine—are we there yet? A narrative review of precision medicine’s applicability in primary care. J Pers Med. (2024) 14:418. 10.3390/jpm14040418 38673045 PMC11051552

[3] Taylor DJ Eizenga JM Li Q Das A Jenike KM Kenny EE Beyond the human genome project: The age of complete human genome sequences and pangenome references. Annu Rev Genomics Hum Genet. (2024) 25(1):77–104. 10.1146/annurev-genom-021623-081639 38663087 PMC11451085

[4] Rodríguez SM Rodríguez-Hernández AM Torres-Torres G Centeno-Girona H Cruz-Correa M. Tumor molecular profiling in hispanics: moving towards precision oncology and health equity. J Racial Ethn Heal Disparities. (2023) 10:1423–31. 10.1007/s40615-022-01328-0 PMC10163076 35648382

[5] Reedy J Blanchard JW Lund J Spicer PG Byars C Peercy M Deliberations about genomic research and biobanks with citizens of the chickasaw nation. Front Genet. (2020) 11:466. 10.3389/fgene.2020.00466 32477408 PMC7240027

[6] Abadie R Heaney K. “We can wipe an entire culture”: fears and promises of DNA biobanking among native Americans. Dialect Anthr. (2015) 39:305–20. 10.1007/s10624-015-9391-4

[7] Walsh SJ Mitchell RJ Curran JM Buckleton JS. The extent of substructure in the indigenous Australian population and its impact on DNA evidence interpretation. Int Congr Ser. (2006) 1288:382–4. 10.1016/j.ics.2005.09.075

[8] Mychaleckyj JC Havt A Nayak U Pinkerton R Farber E Concannon P Genome-wide analysis in brazilians reveals highly differentiated native American genome regions. Mol Biol Evol. (2017) 34:msw249. 10.1093/molbev/msw249 PMC5430616 28100790

[9] Which population is most genetically distant from Africans? Amerindians | Discover Magazine. Available online at: https://www.discovermagazine.com/which-population-is-most-genetically-distant-from-africans-amerindians-32883 (Accessed September 15, 2025).

[10] Lyeo JS Liberda EN Ahmed F Charania NA Moriarity RJ Tsuji LJ Recognising the heterogeneity of indigenous peoples during the COVID-19 pandemic: a scoping review across Canada, Australia, New Zealand and the USA. BMJ Public Heal. (2024) 2:e001341. 10.1136/bmjph-2024-001341 PMC11816692 40018612

[11] Radin J. “Digital natives”: how medical and indigenous histories matter for big data. Osiris. (2017) 32:43–64. 10.1086/693853

[12] Marley TL. Indigenous data sovereignty: university institutional review board policies and guidelines and research with American Indian and Alaska native communities. Am Behav Sci. (2019) 63:722–42. 10.1177/0002764218799130

[13] Credo J Ingram JC. Perspective developing successful collaborative research partnerships with AI/AN communities. Int J Environ Res Public Heal. (2021) 18:9089. 10.3390/ijerph18179089 PMC8430766 34501677

[14] Hiratsuka VY Beans JA Reedy J Yracheta JM Peercy MT Saunkeah B Fostering ethical, legal, and social implications research in tribal communities: the center for the ethics of indigenous genomic research. J Empir Res Hum Res Ethics. (2020) 15:271–8. 10.1177/1556264619872640 31496352 PMC7061084

[15] Fox K. The illusion of inclusion—the “all of us” research program and indigenous peoples’ DNA. N Engl J Med. (2020) 383:411–3. 10.1056/NEJMp1915987 32726527

[16] Pasquetto I Cullen Z Thomer A Wofford M. Open research data poses real world risks that need to be managed—Impact of Social Sciences. Available online at: https://blogs.lse.ac.uk/impactofsocialsciences/2024/11/19/open-research-data-poses-real-world-risks-that-need-to-be-managed/ (Accessed September 15, 2025).

[17] Ugochukwu AI Phillips PWB. Open data ownership and sharing: challenges and opportunities for application of FAIR principles and a checklist for data managers. J Agric Food Res. (2024) 16:101157. 10.1016/j.jafr.2024.101157

[18] Grubb AM Easterbrook SM. On the lack of consensus over the meaning of openness: an empirical study. PLoS One. (2011) 6:e23420. 10.1371/journal.pone.0023420 21858110 PMC3157385

[19] A brief history of Open Data. Available online at: https://www.paristechreview.com/2013/03/29/brief-history-open-data/ (Accessed September 15, 2025).

[20] Hu C Li C Zhang G Lei Z Shah M Zhang Y CrowdMed-II: a blockchain-based framework for efficient consent management in health data sharing. World Wide Web. (2022) 25:1489–515. 10.1007/s11280-021-00923-1 35002477 PMC8720166

[21] Sahni NR Carrus B. Artificial intelligence in U.S. Health care delivery. N Engl J Med. (2023) 389:348–58. 10.1056/NEJMra2204673 37494486

[22] Carroll SR Garba I Figueroa-Rodríguez OL Holbrook J Lovett R Materechera S The CARE principles for indigenous data governance. Data Sci J. (2020) 19:43. 10.5334/dsj-2020-043

[23] Zong J Matias JN. Data refusal from below: a framework for understanding, evaluating, and envisioning refusal as design. ACM J Responsible Comput. (2024) 1:1–23. 10.1145/3630107

[24] Mackey TK Calac AJ Chenna Keshava BS Yracheta J Tsosie KS Fox K. Establishing a blockchain-enabled indigenous data sovereignty framework for genomic data. Cell. (2022) 185:2626–31. 10.1016/j.cell.2022.06.030 35868267

[25] Waltz TJ Powell BJ Fernández ME Abadie B Damschroder LJ. Choosing implementation strategies to address contextual barriers: diversity in recommendations and future directions. Implement Sci. (2019) 14:42. 10.1186/s13012-019-0892-4 31036028 PMC6489173

[26] ERIC—eJ1101588—first nations and higher education: the four r’s–respect, relevance, reciprocity, responsibility. J College Univer Student Housing. (2016) 32:94–109. https://eric.ed.gov/?id=EJ1101588 (Accessed September 15, 2025).

[27] Jacobsen A Azevedo RM Juty N. FAIR Principles: interpretations and implementation considerations. Data Intell. (2020) 2:10–29. 10.1162/dint_r_00024

[28] Denter NM Seeger F Moehrle MG. How can blockchain technology support patent management? A systematic literature review. Int J Inf Manag. (2023) 68:102506. 10.1016/j.ijinfomgt.2022.102506

[29] Chambers DA. Advancing adaptation of evidence-based interventions through implementation science: progress and opportunities. Front Heal Serv. (2023) 3:1204138. 10.3389/frhs.2023.1204138 PMC10277471 37342795

[30] Duarte ME Vigil-Hayes M Littletree S. Of course, data can never fully represent reality. Hum Biol. (2020) 91:163–78. 10.13110/humanbiology.91.3.03 32549034

[31] Rowe R Carroll SR Healy C. The SEEDS of indigenous population health data linkage. Int J Popul Data Sci. (2021) 6:1417. 10.23889/ijpds.v6i1.1417 34212119 PMC8218891

[32] James R Tsosie R Sahota P Parker M Dillard D Sylvester I Exploring pathways to trust: a tribal perspective on data sharing. Genet Med. (2014) 16:820–6. 10.1038/gim.2014.47 24830328 PMC4224626

[33] Carroll SR Suina M Jäger MB Black J Cornell S Gonzales AA Reclaiming indigenous health in the US: moving beyond the social determinants of health. Int J Environ Res Public Heal. (2022) 19:7495. 10.3390/ijerph19127495 PMC9223447 35742745

[34] Cresci VL James RD. The role of tribal epidemiology centers in serving the public health needs of American Indians and Alaska natives. J Public Heal Manag Pract. (2019) 25:S1–2. 10.1097/PHH.0000000000001008 Publish Ahead of Print:. 31349346

[35] O’Connell MC Abourezk C. Facilitating the urgent public health need to improve data sharing with tribal epidemiology centers. Public Heal Rep. (2023) 138:80S–3. 10.1177/00333549231152197 PMC10515977 36734206

[36] Carroll SR Akee R Chung P Cormack D Kukutai T Lovett R Indigenous Peoples’ data during COVID-19: from external to internal. Front Sociol. (2021) 6:617895. 10.3389/fsoc.2021.617895 33869569 PMC8022638

[37] Huyser KR Horse AJY Kuhlemeier AA Huyser MR. COVID-19 Pandemic and indigenous representation in public health data. Am J Public Heal. (2021) 111:S208–14. 10.2105/AJPH.2021.306415 PMC8561074 34709868

[38] Arrazola J Masiello MM Joshi S Dominguez AE Poel A Wilkie CM COVID-19 Mortality among American Indian and Alaska native persons—14 states, January–June 2020. Morb Mortal Wkly Rep. (2020) 69:1853–6. 10.15585/mmwr.mm6949a3 PMC7737685 33301432

[39] Rodriguez NM Burleson G Linnes JC. Thinking beyond the device: an overview of human- and equity-centered approaches for improved health technology design. Annu Rev Biomed Eng. (2023) 25:257–80. 10.1146/annurev-bioeng-081922-024834 37068765 PMC10640794

[40] Tamwoy N Rosas S Davis S Farthing A Houghton C Johnston H Co-design with aboriginal and torres strait islander communities: a journey. Aust J Rural Heal. (2022) 30:816–22. 10.1111/ajr.12918 36037400

[41] View of Comparing Federal Indigenous Health Policy Reform in Canada and the United States | Health Reform Observer—Observatoire des Réformes de Santé. https://mulpress.mcmaster.ca/hro-ors/article/view/5094/4309 (Accessed September 15, 2025).

[42] Hudson M Carroll SR Anderson J Blackwater D Cordova-Marks FM Cummins J Indigenous Peoples’ rights in data: a contribution toward indigenous research sovereignty. Front Res Metr Anal. (2023) 8:1173805. 10.3389/frma.2023.1173805 37215248 PMC10192690

[43] Bowen J Hinze A. Participatory data design: managing data sovereignty in IoT solutions. Interact Comput. (2022) 34:60–71. 10.1093/iwc/iwac031

[44] Indigenous Data Sovereignty Toward and Agenda. Available online at: https://library.oapen.org/bitstream/handle/20.500.12657/31875/624262.pdf (Accessed September 15, 2025).

[45] Claw KG Anderson MZ Begay RL Tsosie KS Fox K Garrison N’A A framework for enhancing ethical genomic research with indigenous communities. Nat Commun. (2018) 9:2957. 10.1038/s41467-018-05188-3 30054469 PMC6063854

[46] Prictor M Teare HJA Kaye J. Equitable participation in biobanks: the risks and benefits of a “dynamic consent” approach. Front Public Heal. (2018) 6:253. 10.3389/fpubh.2018.00253 PMC6133951 30234093

[47] Lo K Liu F Huang J. Onefeather Mobile wallet: a digital solution for indigenous peoples in Canada? Account Perspect. (2021) 20:403–19. 10.1111/1911-3838.12263

[48] al Kez D Foley AM Laverty D Del Rio DF Sovacool B. Exploring the sustainability challenges facing digitalization and internet data centers. J Clean Prod. (2022) 371:133633. 10.1016/j.jclepro.2022.133633

[49] Siddik MAB Shehabi A Marston L. The environmental footprint of data centers in the United States. Environ Res Lett. (2021) 16:064017. 10.1088/1748-9326/abfba1

[50] rodriguez-Lonebear D Barceló NE Akee R Carroll SR. American Indian reservations and COVID-19: correlates of early infection rates in the pandemic. J Public Heal Manag Pr. (2020) 26:371–7. 10.1097/PHH.0000000000001206 PMC7249493 32433389

[51] mcinnes G Sharo AG Koleske ML Brown JEH Norstad M Adhikari AN Opportunities and challenges for the computational interpretation of rare variation in clinically important genes. Am J Hum Genet. (2021) 108:535–48. 10.1016/j.ajhg.2021.03.003 33798442 PMC8059338

[52] Cummings BB Marshall JL Tukiainen T Lek M Donkervoort S Foley AR Improving genetic diagnosis in Mendelian disease with transcriptome sequencing. Sci Transl Med. (2017) 9(386):eaal5209. 10.1126/scitranslmed.aal5209 28424332 PMC5548421

[53] Mersha TB. From Mendel to multi-omics: shifting paradigms. Eur J Hum Genet. (2024) 32:139–42. 10.1038/s41431-023-01420-x 37468578 PMC10853174

[54] Haring RC Blanchard JW Korchmaros JD Lund JR Haozous EA Raphaelito J Empowering equitable data use partnerships and indigenous data sovereignties amid pandemic genomics. Front Public Heal. (2021) 9:742467. 10.3389/fpubh.2021.742467 PMC8632014 34858924

[55] Morton DJ Proudfit J Calac D Portillo M Lofton-Fitzsimmons G Molina T Creating research capacity through a tribally based institutional review board. Am J Public Heal. (2013) 103:2160–4. 10.2105/AJPH.2013.301473 PMC3828979 24134381

[56] Kuhn NS Parker M Lefthand-Begay C. Indigenous research ethics requirements: an examination of six tribal institutional review board applications and processes in the United States. J Empir Res Hum Res Ethics. (2020) 15:279–91. 10.1177/1556264620912103 32233729

[57] Saldana L Ritzwoller DP Campbell M Block EP. Using economic evaluations in implementation science to increase transparency in costs and outcomes for organizational decision-makers. Implement Sci Commun. (2022) 3:40. 10.1186/s43058-022-00295-1 35410434 PMC9004101

[58] Haozous EA Lee J Soto C. Urban American Indian and Alaska native data sovereignty: ethical issues. Am Indian Alsk Nativ Ment Heal Res. (2021) 28:77–97. 10.5820/aian.2802.2021.77 PMC8877071 34586627

[59] Damschroder LJ Reardon CM Widerquist MAO Lowery J. The updated consolidated framework for implementation research based on user feedback. Implement Sci. (2022) 17:75. 10.1186/s13012-022-01245-0 36309746 PMC9617234

[60] Delaforce A Li J Grujovski M Parkinson J Richards P Fahy M Creating an implementation enhancement plan for a digital patient fall prevention platform using the CFIR-ERIC approach: a qualitative study. Int J Environ Res Public Heal. (2023) 20:3794. 10.3390/ijerph20053794 PMC10001076 36900804

[61] Public deliberation | Involve. Available online at: https://www.involve.org.uk/resources/knowledge-base/what/public-deliberation (Accessed September 15, 2025).

[62] Jensen M Verhagen H Vanhée L Dignum F. Advances in social simulation, in Proceedings of the 16th Social Simulation Conference, 20–24 September 2021. Springer Proc Complex (2022). p. 409–21.

[63] Hunt SC Young NL. Blending indigenous sharing circle and western focus group methodologies for the study of indigenous children’s health: a systematic review. Int J Qual Methods. (2021) 20:16094069211015112. 10.1177/16094069211015112

[64] makosky Daley C James AS Ulrey E Joseph S Talawyma A Choi WS Using focus groups in community-based participatory research: challenges and resolutions. Qual Heal Res. (2010) 20:697–706. 10.1177/1049732310361468 PMC2947156 20154299

[65] Ardoin NM Gould RK Wojcik D Wyman Roth N Biggar M. Community listening sessions: an approach for facilitating collective reflection on environmental learning and behavior in everyday life. Ecosyst People. (2022) 18:469–77. 10.1080/26395916.2022.2101531

[66] Sebastian S Thomas DP Brimblecombe J Majoni V Cunningham FC. Factors impacting on development and implementation of training programs for health professionals to deliver brief interventions, with a focus on programs developed for indigenous clients: a literature review. Int J Environ Res Public Heal. (2020) 17:1094. 10.3390/ijerph17031094 PMC7037654 32050440

[67] Damschroder LJ Reardon CM Opra Widerquist MA Lowery J. Conceptualizing outcomes for use with the consolidated framework for implementation research (CFIR): the CFIR outcomes addendum. Implement Sci. (2022) 17:7. 10.1186/s13012-021-01181-5 35065675 PMC8783408

[68] Calac AJ Mackey TK. A systematic review of responsible stewardship of research and health data from indigenous communities. NPJ Digit Med. (2025) 8(1):504. 10.1038/s41746-025-01902-w 40764752 PMC12326002

[69] Carroll SR Plevel R Jennings LL Garba I Sterling R Cordova-Marks FM Extending the CARE principles from tribal research policies to benefit sharing in genomic research. Front Genet. (2022) 13:1052620. 10.3389/fgene.2022.1052620 36437947 PMC9691892

[70] Soltoff AE Isaacson MJ Stoltenberg M Duran T Laplante L“JR” Petereit D Utilizing the consolidated framework for implementation research to explore palliative care program implementation for American Indian and Alaska natives throughout the United States. J Palliat Med. (2022) 25:643–9. 10.1089/jpm.2021.0451 35085000 PMC12396782

[71] Garrison N'A Barton KS Porter KM Mai T Burke W Carroll SR. Access and management: indigenous perspectives on genomic data sharing. Ethn Dis. (2019) 29:659–68. 10.18865/ed.29.S3.659 31889771 PMC6919970

